# Case of inflammatory granuloma in inguinal hernia sac after hysterosalpingography with oily contrast medium

**DOI:** 10.1016/j.ijscr.2020.05.084

**Published:** 2020-06-09

**Authors:** Yoshiko Miyazaki, Tetsu Yamamoto, Ryoji Hyakudomi, Takahito Taniura, Takanori Hirayama, Kiyoe Takai, Noriyuki Hirahara, Yoshitsugu Tajima

**Affiliations:** a Department of Digestive and General Surgery, Shimane University, Faculty of Medicine, Japan; b National Hospital Organization, Nagasaki Medical Center, Japan

**Keywords:** Inflammatory granuloma, Hysterosalpingography, Oily contrast medium, Inguinal hernia

## Abstract

**Introduction:**

The foreign body reaction caused by oil contrast medium, Lipiodol, is rare. We present a rare case of inflammatory granuloma in the inguinal hernia sac after hysterosalpingography with lipiodol.

**Presentation of case:**

A 30-year-old woman who had left inguinal growing mass for 7 months after hysterosalpingography with Lipiodol for examination of infertile. About 3 cm soft mass was palpable on the left inguinal region, and plane CT scan showed a multifocal cystic mass with metal concentration. Thus, laparoscopic herniorrhaphy was performed to repair the inguinal hernia and to confirm the contents of cystic lesion. The hernia sac filled with mucinous contents. Pathological examination revealed that inflammatory granuloma due to numerous lipid droplets and phagocytic images of leukocytes in the hernia capsule.

**Discussion and conclusion:**

Although oil contrast medium is safety agent, there is rear complication which need the surgical treatment. Complete resection of granuloma, including hernia sac, is important to prevent recurrence.

## Highlights

• Oil contrast medium caused inflammatory granuloma.
• Inflammatory granuloma caused growing hernia as the contents of inguinal hernia.
• Complete resection of granuloma, including hernia sac, is important to prevent recurrence.

## 1 Introduction

Lipiodol is now widely used as a contrast agent in the clinical situation. Especially, improvements of fertility after hysterosalpingography using lipiodol were reported in the field of obstetrics and gynecology [1]. However, some papers reported about the foreign body reaction after contrast examination using Lipiodol, because it is an oil contrast agent and tend to remain in the body for long time [2]. In addition, some deuteropathies which require the any treatment (e.g. pelvic or diaphragmatic cysts, stricture of the intestine, the bile duct and the ureter) are easy to occur when the inflammatory granuloma exist in the intraperitoneally after hysterosalpingography.

We reported a rear case of inflammatory granulomatous alteration in the inguinal hernia sac by accumulation of Lipiodol after hysterosalpingography, who was diagnosed and treated by laparoscopic surgery. This article has been written according to SCARE criteria [3].

## 2 Presentation of case

A 30-year-old woman with left inguinal growing mass consulted our hospital.

**Fig. 1:**
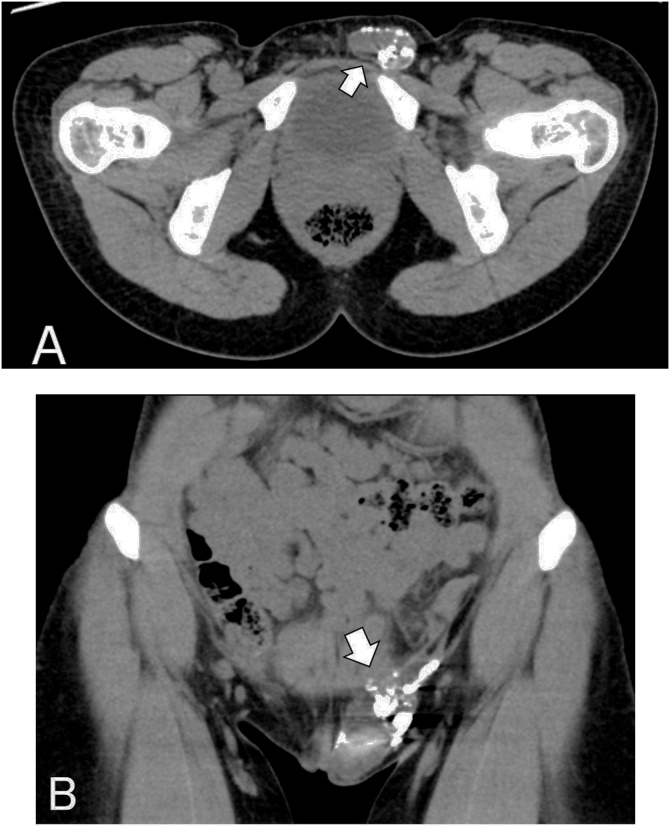
CT scan showed a cystic mass with scattered metal artifacts on the left inguinal region. The max CT value of artifacts was 6000–8000 H.U. a: axial view, b: coronal view.

She was examined by hysterosalpingography with Lipiodol about 8 months before consult because of infertile at another hospital. She recognized left inguinal mass 7 months after the examination. About 3 cm of soft mass was palpable on the left inguinal region in a standing position, and this mass was disappeared in a spine position. The patient was afebrile and the blood tests revealed neither neutrophilic leukocytosis nor raised level of inflammatory C-reactive protein. Plane CT scan showed a multilocular mass in the left inguinal region (Fig. 1A and B). The max CT value was 6000–8000 H.U., indicated that the content was oil contrast medium or metal concentration. No retention of contrast medium was observed in the uterus or around the appendage. Hence, it was strongly suggested that the contrast medium by hysterosalpingography stored in the hernia sac through hernia orifice, and the contrast medium was pooled in hernia sac, because the hernia orifice was too narrow.

Trans vaginal ultrasonography showed bilateral ovaries were present in the pelvis and these were normal. Some aberrations such as endometriosis, hydrocele of the canal of Nuck, etc. were considered as differential diagnosis. Thus, we decided to perform a surgical treatment, laparoscopic hernia repair with hernia sac resection, was mandatory.

## 3 Surgical findings

**Fig. 2:**
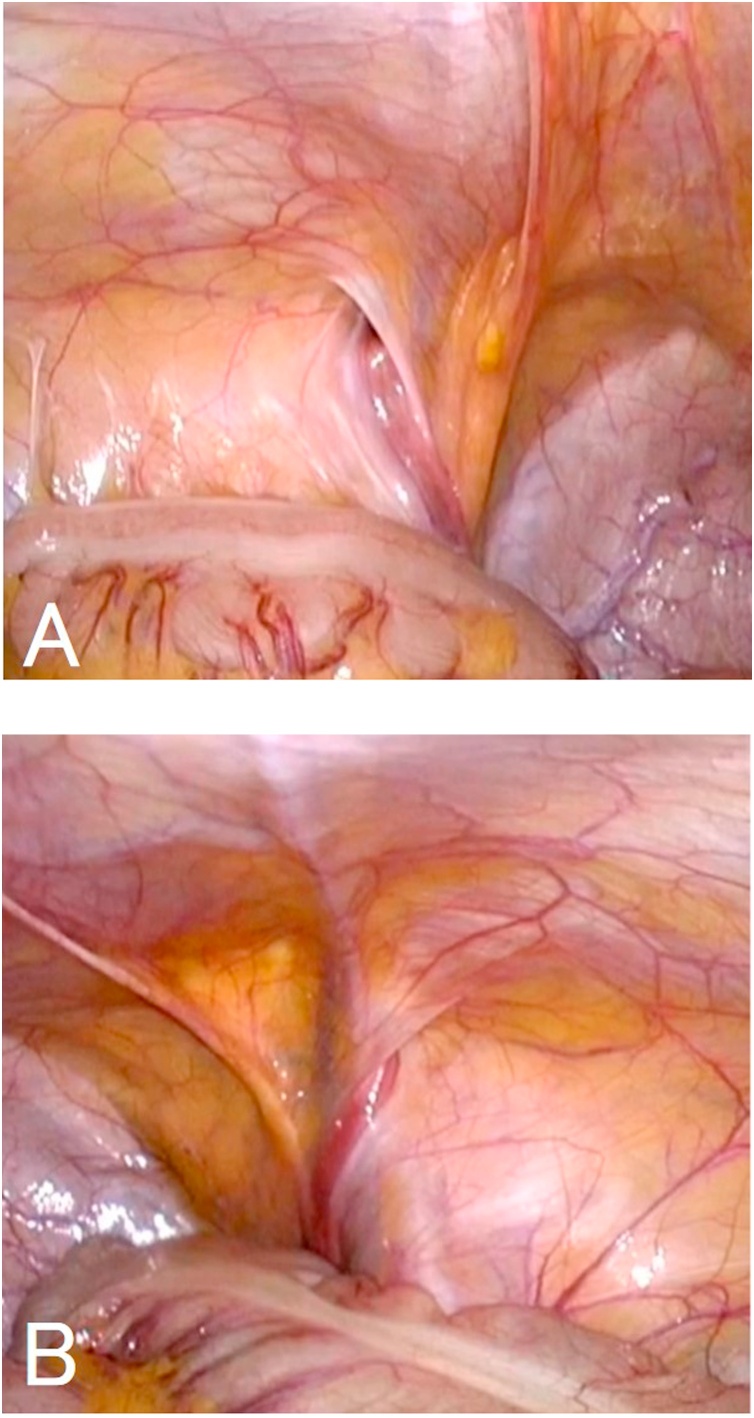
Laparoscopic view of bilateral indirect inguinal hernias. a: left inguinal hernia (type I-1 of Japan Hernia Society classification) b: right inguinal hernia (type I-1 of Japan Hernia Society classification).

**Fig. 3:**
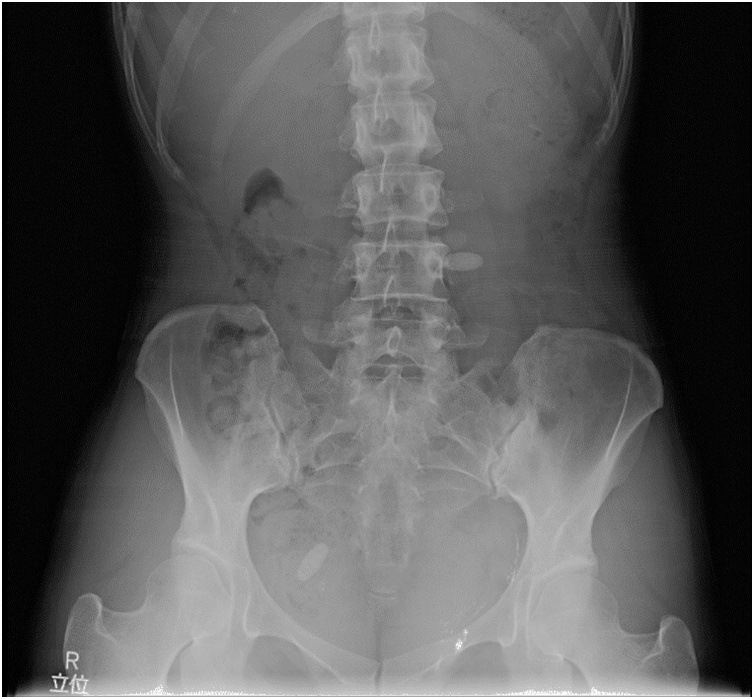
An abdominal X-ray examination after laparoscopic inguinal herniorrhaphy. No dense materials in the peritoneal cavity was recognized.

About 2 cm of skin incision was made on the umbilicus, and a camera port was inserted by the open laparotomy method. Bilateral indirect inguinal hernias (I-1 of The Japan Hernia Society (JHS) Classification for groin hernia) were recognized (Fig. 2A and B). A 5 mm of working port was inserted at the height of the umbilicus of the right abdomen, and 3 mm of Endo Relief (Hope Denshi Co., Ltd, Kamagaya, JAPAN) was inserted at the same height of the left abdomen. When the hernia of the left inguinal region was pulled out, a cystic lesion was recognized at the caudal side of hernia sac. An incision was made in the peritoneum of the hernia orifice and the cyst was isolated and resected with hernia sac. For right side of inguinal hernia, LPEC (Laparoscopic percutaneous extraperitoneal closure) using 2-0 ETHIBOND EXCEL® (Ethicon Inc. Somerville, NJ) was performed, because the remnant vaginal process of peritoneum was present. The patients had a good clinical course and she was discharged after 2 days after operation. The high absorption region was not recognized by abdominal X-ray photography. The patient had no signs of recurrence were observed 2 year after surgery (Fig. 3).

## 4 Pathological findings

**Fig. 4:**
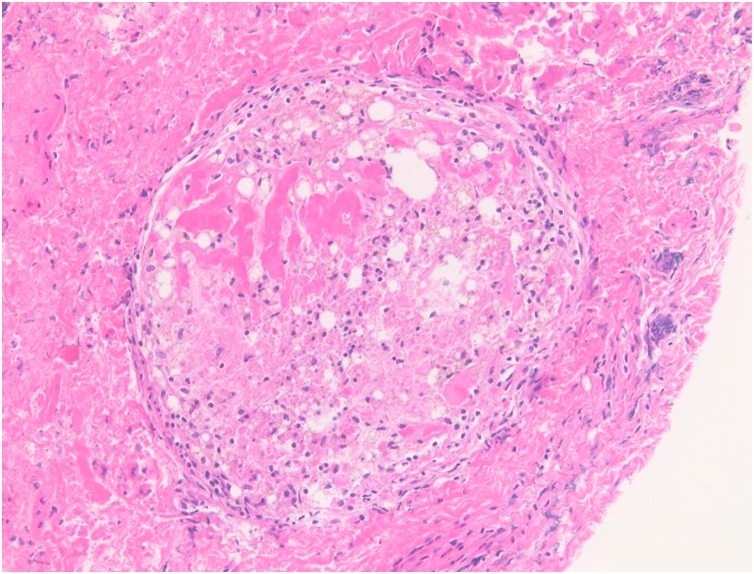
Pathological findings of the inguinal hernia sac. Thickened fibrous capsule with fibroblasts proliferation and foal collection of foam cells were observed.

The hernia sac was generally fibrously thickened cyst, and the increase of fibroblasts and the collection area of foam cells were observed (Fig. 4). It was a consistent finding as inflammatory granuloma by oil contrast medium, Lipiodol.

## 5 Discussion

Hysterosalpingography is a basical examination method of infertility, a method of injecting a contrast medium from the vagina side into the lumen of the uterus, observing the shape of the uterine cavity and checking the passability of the fallopian tubes. There are two type of contrast medium, oily and aqueous. And oily contrast agents are widely used because of their superiority of rendering properties and the high possibility of pregnancy after treatment. Lipiodol, an oily contrast agent, is more widely used as a contrast agent than aqueous contrast because of its less complication rate, such as systemic reaction and peritoneal irritation. However, there is a few reports that oily contrast medium is hardly absorbed in the body and remains in the long term, resulting in inflammatory granuloma due to foreign body reaction [4].

Inflammatory granuloma is one of the chronic inflammation caused by accumulation of inflammatory cells, such as macrophage, phagocytosing lipids, epithelial cells, multinucleated giant cells. Many papers reported that inflammatory granuloma is easily caused in the kidney or gall bladder [5]. However, there is no report about inflammatory granuloma in the hernia sac.

In this case, the patient had no symptom of groin hernia before hysterosalpingography, and it takes about 7 months when the patient recognized groin mass on the left inguinal region. Thus, it is speculated that Lipiodol which remain in hernia sac for a long time cause foreign body reaction, resulting in inflammatory granuloma.

Although there are many reports that surgical resection was performed as a diagnostic treatment for the inflammatory granuloma, no standard therapy was established. In this case we underwent inguinal hernia repair by the transabdominal preperitoneal approach (TAPP). There are two reasons, 1) the form and properties of cystic lesions can be confirmed from intraabdominal cavity, 2) whole abdomen, especially pelvic cavity, and bilateral inguinal hernia are able to be observed. Some papers reported that the cystic formation with inflammatory granuloma on the pelvis, diaphragm and intestinal tract after uterine oviducture contrast examination caused stricture of the ureter, bile duct and intestine [[5], [6], [7]]. Thus, observation of the entire abdominal cavity during laparoscopic surgery is very important and considered to be a great advantage.

We performed herniorrhaphy by LPEC for the contra lateral inguinal hernia which was no symptom in this case, because there was a possibility that the intra-abdominal pressure will be rise when she will pregnant, and contralateral inguinal hernia will be occurred. Although regarding the adequacy of LPEC for adults was controversial, Because the mechanism of occurrence of young adult inguinal hernia is often the same as in childhood, treatment with high - level ligation method can be considered. Recently, there are some papers reported that LPEC is effective for a inguinal hernia of a young woman without vulnerability of the abdominal wall [[8], [9], [10]]. Thus, LPEC has benefit of minimizing insertion of foreign body. However, farther investigations are needed to evaluate the effect of LPEC on the adult inguinal hernia.

## 6 Conclusion

We reported the rare case of inguinal hernia due to inflammatory granuloma by oil contrast medium. Although oil contrast medium is safety agent, there is rear complication which need the surgical treatment. Complete resection of granuloma, including hernia sac, is important to prevent recurrence.

## Declaration of Competing Interest

All authors declare no conflicts of interest associated with this manuscript.

## Sources of funding

This research did not receive any specific grant from funding agencies in the public, commercial, or not-for-profit sectors.

## Ethical approval

The study was reviewed and approved by the Shimane University Institutional Review Board. The reference number is 20200127-1.

## Consent

Informed consent was obtained from the patient.

## Author contribution

Contributors YT was responsible for the organisation and coordination of this study. TY was the chief investigator and also responsible for the data analysis. RH, TT, TH and NH planned this study. All authors contributed to the writing of the final manuscript. All authors contributed equally to the conception, design, literature review, analysis, drafting, critical revision, and editing and approved the final version of this study.

## Registration of research studies

This case report was registered at http://www.researchregistry.com. The identifying number is researchregistry5607.

## Guarantor

Tetsu Yamamoto and Yoshitsugu Tajima.

## Institutional review board statement

The study was reviewed and approved by the Shimane University Institutional Review Board.

## Provenance and peer review

Not commissioned, externally peer-reviewed.
